# Genetic Variants of *MIR27A*, *MIR196A2* May Impact the Risk for the Onset of Coronary Artery Disease in the Pakistani Population

**DOI:** 10.3390/genes13050747

**Published:** 2022-04-24

**Authors:** Taqweem Ul Haq, Abdul Zahoor, Yasir Ali, Yangchao Chen, Fazal Jalil, Aftab Ali Shah

**Affiliations:** 1 Department of Biotechnology, University of Malakand, Chakdara 18000, Pakistan; taqweembiotech@yahoo.com (T.U.H.); zahoor868@gmail.com (A.Z.); 2 Department of Biotechnology, Abdul Wali Khan University Mardan (AWKUM), Mardan 23200, Pakistan; yasirali@awkum.edu.pk (Y.A.); fazaljalil@awkum.edu.pk (F.J.); 3 School of Biomedical Sciences, The Chinese University of Hong Kong, Shatin, Hong Kong; yangchaochen@cuhk.edu.hk

**Keywords:** allele-specific PCR, CAD, DNA, miRNA, molecular marker, SNP

## Abstract

Genetic variants in microRNA genes have a detrimental effect on miRNA-mediated regulation of gene expression and may contribute to coronary artery disease (CAD). CAD is the primary cause of mortality worldwide. Several environmental, genetic, and epigenetic factors are responsible for CAD susceptibility. The contribution of protein-coding genes is extensively studied. However, the role of microRNA genes in CAD is at infancy. The study is aimed to investigate the impact of rs895819, rs11614913, and rs2168518 variants in *MIR27A*, *MIR196A2*, and *MIR4513*, respectively, in CAD using allele-specific PCR. Results: For variant rs11614913, significant distribution of the genotypes among the cases and controls was determined by co-dominant [χ^2^ = 54.4; *p* value ≤ 0.0001], dominant (C/C vs. C/T + T/T) [OR = 0.257 (0.133–0.496); *p* value ≤ 0.0001], recessive (T/T vs. C/T + C/C) [OR = 1.56 (0.677–0.632); *p* value = 0.398], and additive models [OR = 0.421 (0.262–0.675); *p* value = 0.0004]. Similarly, a significant association of rs895819 was determined by co-dominant [χ^2^ = 9.669; *p* value ≤ 0.008], dominant (A/A vs. A/G + G/G) [OR = 0.285 (0.1242–0.6575); *p* value ≤ 0.0034], recessive (G/G vs. A/G + A/A) [OR = 0.900 (0.3202–3.519); *p* value = 1.000], and additive models [OR = 0.604 (0.3640–1.002); *p* value = 0.05] while no significant association of rs2168518 with CAD was found. Conclusion: The variants rs895819 and rs11614913 are the susceptibility factors for CAD.

## 1. Introduction

MicroRNAs (miRNAs) are a group of small non-coding RNAs molecules (20–24 nucleotides) that repress mRNAs post-transcriptionally [1,2,3,4]. MicroRNA regulates the biological phenomenon at the post-transcriptional level [5]. MiRNAs play a pivotal role in the post-transcriptional regulation of protein-coding genes [6]. Dysregulation of miRNA expression is connected with several human diseases including coronary artery disease (CAD) [7]. Since the first discovery of miRNA, there are 38,589 entries representing hairpin precursor microRNAs, from 271 organisms. The human genome contains 1917 annotated hairpin precursors and 2654 mature sequences [8]. The seed region comprises 2–8 nucleotides of the miRNA and the recognition of the target genes depends mainly on their pairing [9]. Target miRNA cleavage or translational inhibition are two independent ways of miRNA-mediated translational control [10]. About half of miRNAs are transcribed from introns (intragenic) and relatively few are from exons of protein-coding genes. Similarly, few are intergenic and transcribed independently [11,12,13] while some miRNAs are located as clusters and their transcription has resulted in one long transcript [14]. However, the interaction of miRNAs with other regions in the target mRNA including the 5′-untranslated regions, coding sequence, and gene promoters, have also been reported [15]. The miRNAs lead the argonaut proteins to specific target messenger RNAs to suppress their stability and translation.

Aberrant miRNA expression is mostly due to single nucleotide polymorphism (SNP) in miRNA genes [16]. SNP is the most common form of single base-pair changes in a genome. The previous study has shown that SNPs have a profound influence on miRNA function, stability, and targeting [17]. SNPs are commonly identified in the miRNA genes or the binding site of mRNA of miRNAs. SNPs in the miRNA binding site can modify miRNA by generating or removing a miRNA binding site in the target mRNA [18,19]. Several studies showed that SNPs in the target site of miRNA genes are involved in a wide range of diseases [7,20,21]. It was investigated that the SNP (rs531564) is associated with an increased risk of cervical cancer, diabetic retinopathy, and CAD [22,23,24]. Coronary artery disease (CAD) is a pathological condition linked with atherosclerotic plaque aggregation in the epicardial arteries, whether obstructive or non-obstructive [25]. The association of mir-499-SNP (rs3746444) with ischemic stroke in the Asian population was reported [26]. It was found that miRNAs play a role in the pathophysiology of Cardiomyopathy [27]. The core heptameric sequence of a mature miRNA term as “seed region,” includes 2–8 nucleotides and plays a crucial role in target gene recognition and interaction [4,28]. Genetic variations in the seed regions have crucial impacts on gene expression and disease susceptibility in humans [1]. The distribution pattern of genetic variation in miRNA seed regions might be related to miRNA function [2,3,29].

A previous study indicates that CAD leads to one-third of mortality in women regardless of their ethnicity [30]. An earlier study showed that more than six percent of the adult population is suffering from CAD [31]. Atherosclerosis is a condition in which plaque builds up inside the arteries that provide oxygenated blood to the heart and are leading to CAD [32,33]. The plaque is made over the years that lead to narrowing of the coronary artery lumen. Consequently, limits the flow of blood to the artery [34]. Several studies have shown the contribution of alteration in protein-coding genes with CAD [35,36]. However, the association of genetic alteration in non-coding genomes especially microRNA genes, and pathophysiology of CAD is investigated very rarely [37,38,39]. Mature microRNA plays a pivotal role in the endothelial function in cardiometabolic disorders. In CAD patients, miR-206 has been demonstrated to reduce the viability and invasion of endothelial progenitor cells while increasing their death [25,40,41,42,43]. A previous study has shown that miR-92a-3p is up-regulated in CAD [44]. It was also noted high expression of miR-330 blocked the formation of plaques in atherosclerosis [45].

It was also noted that MiR-1 plays a pivotal role in the progression of CAD, as well as in cardiogenesis and cardiac hypertrophy. It was investigated that miR-1 is upregulated in CAD patients through regulation of cardiac arrhythmogenic potential by targeting several ion channel genes [46,47]. Interestingly it was noted that miR-322 was upregulated in unstable plaques compared to stable plaques that are formed during CAD progression [48]. The current study is focused on the genetic alteration in microRNA (miRNA) genes and their association with CAD in the Pakistani population. Therefore, SNPs in the miRNA seed region are likely to change the target genes expression and influence the corresponding phenotypes.

## 2. Materials and Methods

### 2.1. Study Population 

This current case-control study was designed to investigate the genetic-based risk factors for CAD. In this study, we examined 223 coronary artery disease individuals (CAD), as well as 150 healthy controls as shown in Table 1. The patients were clinically diagnosed by a certified cardiologist. A properly designed questionnaire was used to record the demographic and clinical data of each participant and informed consent was obtained from all participants or their guardians. 

### 2.2. Inclusion and Exclusion Criteria for CAD Patients and Healthy Controls

This study was conducted on clinically confirmed cases of CAD patients and healthy controls with no history of CAD. Those CAD patients were selected who had visited a hospital for the evaluation of stable chest pain by elective angiography, electrocardiogram (ECG or EKG), and/or echocardiogram (echo). Patients with a previous history of any chronic disease or those who had performed coronary bypass surgery were excluded from the current study. 

Relevant biochemical tests were also performed to confirm the previous history of CAD. Those healthy controls who have previous cardiac/angina and/or myocardial infarction history were excluded.

### 2.3. Blood Samples Collection and Genomic DNA Extraction

About 3–5 mL of whole blood was collected from February 2019 till January 2020 in EDTA tubes from all patients and healthy controls from District Head Quarter (DHQ) Hospitals of Dir lower and Malakand, Khyber Pakhtunkhwa, Pakistan. DNA was isolated by Phenol/Chloroform method, dissolved in distilled water, and stored at +4 °C until further processing. The quality of extracted DNA was confirmed by a spectrophotometer (Evolution 300 BB, Ser No. EV3 131505, Thermo ELECTRON CORPORATION, Waltham, MA, USA).

### 2.4. Primers for Allele-Specific PCR and Genotyping of Rs895819, Rs11614913, and Rs2168518

The previously published allele-specific PCR primers were used for genotyping of rs895819, rs11614913, and rs2168518 [49,50,51]. PCR products were run on 2% agarose gel and the nature of each genotype (homozygous/heterozygous) was recorded using the visual inspection method of the gel.

### 2.5. Statistical Analysis

Chi-square analysis and Fisher exact test were used to calculate allelic and genotypic frequencies of rs895819, rs11614913, and rs2168518 variants in *MIR27A*, *MIR196A2*, and *MIR4513,* respectively, for both CAD patients and healthy controls. The associations between the studied SNPs and the risk of CAD patients were estimated by calculating the odds ratios (ORs), and with 95% confidence intervals (CIs). GraphPad Prism 6 was used for data analysis.

### 2.6. In-Silico Analyses of the Primary Structure of miRNAs

**Figure 1:**
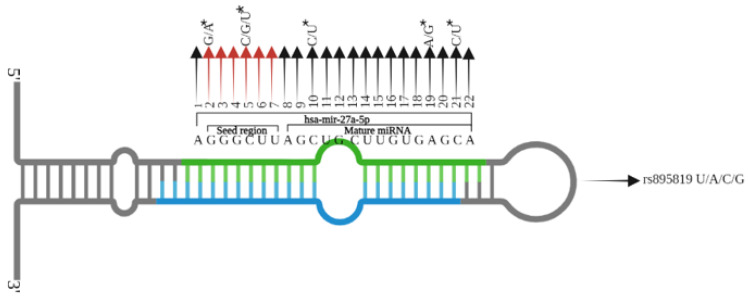
The up-ward arrows show the mature sequence of hsa-mir-27a-3p while the red arrows indicate the seed region (6 nts). Stare (*) Indicates the location of all SNPs in hsa-mir-27a-5p.

Detailed information about the screened rs895819, rs11614913, and rs2168518 in *MIR27A*, *MIR196A2*, and *MIR4513*, their approved gene names, mature miRNAs, location (coordinates) on their respective chromosomes, coded alleles, another allele, and minor allele frequencies are shown in Table 2. Figure 1 shows the locations of the seed region and rs895819 in hsa-mir-27a-3p as an example. To find out whether the rs895819, rs11614913, and rs2168518 variations have some impact on the primary structures of miR-27a, miR-196a2, and miR-4513. The energies of the RNA sequences were determined using the RNAeval web server. Analysis of RNA secondary and centroid structures was carried out with the help of the Vienna RNA website [52]. 

## 3. Results

### 3.1. Association of Rs2168518, Rs895819, and Rs11614913 with CAD 

The current study showed that rs11614913 and rs895819 is linked with the risk of CAD by using different inheritance models as shown in Table 3. The rs11614913 and rs895819 were assessed through different statistical models such as co-dominant, homozygous dominant, homozygous recessive, and additive models. The rs895819 genotypes showed significant association in co-dominant model [χ^2^ = 54.4; *p* < 0.0001], homozygous dominant model [OR = 0.257 (0.133–1.496); *p* < 0.0001], and additive model [OR = 0.421 (0.262–0.675); *p* < 0.0004], but the association of its genotypes was insignificant at homozygous recessive model [OR = 0.156 (0.677–0.632); *p* = 0.398]. Likewise, significant association of rs11614913 with CAD was noted using co-dominant model [χ^2^ = 9.669; *p* < 0.008], homozygous dominant model [OR = 0.285 (0.1242–0.6575); *p* < 0.0034] and additive model [OR = 0.604 (0.364–1.002); *p* = 0.05].

### 3.2. Consequences of Variant Rs2168518, Rs895819, Rs11614913 on miRNA Structure and Properties

Since the variant rs2168518 is in the seed region of *MIR4513*, therefore, there is the substitution of Cytosine with Uracil at position 18 in the mature sequence of hsa-mir-4513-3p. This change leads to an increase in the primary miRNA loop structure by one base pair. By comparing, the normal and mutant primary miRNA structure using the thermodynamic Structure Prediction tool, alterations were also observed in the studied parameters as shown in Table 4. It was observed that the variant rs11614913 in *MIR196A2* resulted in the substitution of Cytosine base by Uracil in the miRNA primary structure. Similarly, due to rs895819, there are 13 bases in the terminal loop structure of wild-type miR-27a centroid structure while 15 bases in the corresponding terminal loop structure of the mutant miR-27a.

## 4. Discussion

It is already known that several environmental, genetic, as well as epigenetic factors, are responsible for the pathophysiology of CAD. Arterial thrombosis is one of the main causes of CAD. Arterial thrombosis is characterized by the development of a blood clot in an artery. Blood vessel occlusion usually occurs when the erosion of associated arterial sclerosis plaque causes wounds particularly in tissues with a terminal tube bed [53]. Arterial sclerosis plaque severely influences the local hemodynamics in coronary arteries [54,55]. The previous study has indicated that the geometry of a plaque is associated with the possibility of clinical incidents, including myocardial infarction in patients with CAD [56]. Coronary plaque geometry is vital for the pathophysiology and enhancing the diagnosis and cure of CAD [57]. There are also serval genetic factors that contribute to arterial thrombosis. Several studies have shown the contribution of SNPs in the protein-coding part of the genome [58,59,60]. Although the role of SNPs in the non-coding part of the genome especially miRNA genes are not fully elucidated. The previous study has shown that SNPs in miRNAs are related to the development of CAD [61]. SNPs located in miRNAs also known as mirSNPs play a pivotal role in the development of various types of diseases. The previous study has explored the role of mirSNPs in CAD in different populations worldwide, however, the role of rs895819, rs11614913, and rs2168518 in CAD in the Pakistani population is in infancy. This study was designed to evaluate the influence of rs895819, rs11614913, and rs2168518 variants located in *MIR27A* and *MIR196A2* and *MIR4513*, respectively, on the risk of CAD in the selected population. The previous study has shown that rs2168518, is significantly linked with blood pressure, LDL, total cholesterol and fasting glucose [49]. It was shown that the expression of miR-4513 is significantly reduced by RNA induced silencing complex (RISC) loading and RNA degradation mechanisms due to the rs2168518 variant in the seed region of *MIR4513* [62,63,64]. Furthermore, it was also investigated that there is a strong association of rs2168518 with the high mortality rate in CAD patients [65]. These data may have significant clinical implications on evaluating the risk of cardiovascular events or the possibility of intensive treatment interventions in CAD patients. The variant rs11614913 is intensively studied in different types of cancers and their association was established in different populations worldwide [66,67,68]. It was investigated that the T allele of the variant rs11614913 C/T in *MIR196A2* was strongly linked with CAD [69]. The current study also confirmed that the T allele of rs11614913 is the risk factor for CAD in the Pakistani Population. The variant rs895819 is one of the broadly studied SNP in different diseases like Type 2 Diabetes Mellitus [70], colorectal cancer [71], and breast cancer [51]. It was also explored that the SNP rs895819 in the primary structure of MIR27A (pre-miR-27a) is associated with susceptibility to myocardial infarction (MI) in the Chinese Hans population. This is following the present study. It is, therefore, recommended that this study should be repeated on a larger cohort. Functional studies of these SNPs should be conducted to investigate their exact role in the pathophysiology of CAD. Furthermore, an expression study should be conducted to provide the basis for the development of blood-borne miRNA-based novel biomarkers for CAD in the future. It is, thus, concluded that these three variants rs895819 and rs11614913 are associated with CAD in the Pakistani population. In the current study, a total of 223 CAD and 150 healthy individuals were genotyped. One of the major limitations of the study is the small sample size. Secondly, the samples may be further validated through the DNA sequencing approach. Moreover, confirmation of these SNPs in CAD patients may be screened on a larger sample size.

## Figures and Tables

**Table 1:** Pre-clinical data about age, gender, BMI, RBS, TC, TG, HDL, and LDL of the CAD patients, as well as healthy controls.

Categories	Age (Year)	Gender	BMI (kg/m^2^)	RBS (mg/dL)	TC (mg/dL)	TG (mg/dL)	HDL (mg/dL)	LDL (mg/dL)
CAD	55.2 (27–91)	Male = 183	23.3 (12.2–38.2)	245.8 (114–415)	222.7 (156–262)	199.3 (110–395)	38.2 (21–58)	126.8 (24–271)
		Female = 40						
Controls	±45	Male = 138	±22.3	±112.4	±200	±162.5	±35	±100
		Female = 12						

BMI: Body mass index, RBS: Random blood sugar, TC: Total cholesterol, TG: Triglyceride, HDL: High-density lipoprotein, LDL: Low-density lipoprotein.

**Table 2:** Shows the list of studied SNPs, their official name, mature miRNA sequences, chromosomal location, and MAF.

SNP ID	miRNA Gene Name	Name of Mature miRNA Sequences	Chromosome No.	miRNA Location (Coordinates)	Coded Allele	Other Alleles	MAF
rs895819	*MIR27A*	hsa-miR-27a-5p	19	13836440-13836517 [−]	T	A/C/G	0.50
		hsa-miR-27a-3p					
rs11614913	*MIR196A2*	hsa-miR-196a-5p	12	53991738-53991847 [+]	C	T	0.49
		hsa-miR-196a-3p					
rs2168518	*MIR4513*	hsa-miR-4513	15	74788672-74788757 [−]	G	A	0.47

**Table 3:** Inheritance models for investigating the association of rs895819, rs11614913, and rs2168518 with CAD.

Gene (Accession Number)	Statistical Models	Genotypes	Cases	Control	Odds Ratiο (95% CI)	χ^2^-Value, df	*p*-Value
*MIR196A2* (rs11614913)	Co-dominant	CC CT TT	24 40 16	50 19 11	—	54.4, 2	<0.0001
	Dominant	CC CT + TT	24 56	50 30	0.257 (0.133–0.496)	—	<0.0001
	Recessive	TT CT + CC	16 64	11 69	1.56 (0.677–0.632)	—	0.398
	Additive	C T	88 72	119 41	0.421 (0.262–0.675)	—	0.0004
*MIR27A* (rs895819)	Co-dominant	AA AG GG	10 46 4	28 35 5	—	9.669, 2	<0.008
	Dominant	AA AG + GG	10 50	28 40	0.285 (0.1242–0.6575)	—	<0.0034
	Recessive	GG AG + AA	4 56	5 63	0.900 (0.3202–3.519)	—	1.000
	Additive	A G	66 54	91 45	0.604 (0.3640–1.002)	—	0.05
*MIR4513* (rs2168518)	Co-dominant	GG GA AA	14 105 24	4 47 19	—	3.682, 2	0.1586
	Dominant	GG GA + AA	14 129	4 66	1.791 (0.5668–5.658)	—	0.4340
	Recessive	AA GA + GG	24 119	19 51	0.5414 (0.2727–1.075)	—	0.1012
	Additive	G A	133 153	55 85	1.343 (0.8905–2.027)	—	0.1773

**Table 4:** Information about the free energy of the thermodynamic ensemble, the frequency of the MFE structure in the ensemble, the ensemble diversity, the optimal secondary structure with minimum free energy, and the centroid secondary structure of studied reference SNPs and their mutated variants.

Parameters	Reference	Mutated	Reference	Mutated	Reference	Mutated
	*MIR4513* rs2168518	*MIR4513* rs2168518	*MIR27A* rs895819	*MIR27A* rs895819	*MIR196A2* rs11614913	*MIR196A2* rs11614913
Free energy of the thermodynamic ensemble	−41.83 kcal/mol	−42.34 kcal/mol	−38.24 kcal/mol	−38.28 kcal/mol.	−52.02 kcal/mol	−46.52 kcal/mol
The frequency of the MFE structure in the ensemble	26.16%	18.55%	15.62%.	14.84%.	6.14%	5.18%
The ensemble diversity	3.58	3.50	4.41	4.55	7.18	7.49
The optimal secondary structure with a minimum free energy	−41.00 kcal/mol	−41.30 kcal/mol	−34.40 kcal/mol	−34.40 kcal/mol	−50.30 kcal/mol	−44.70 kcal/mol
The centroid secondary structure	−41.00 kcal/mol	−41.30 kcal/mol	−37.10 kcal/mol	−37.10 kcal/mol	−49.90 kcal/mol	−44.30 kcal/mol
